# Enthesitis-related arthritis is the most common category of juvenile idiopathic arthritis in Taiwan and presents persistent active disease

**DOI:** 10.1186/s12969-019-0363-0

**Published:** 2019-08-23

**Authors:** Yang-Jen Shih, Yao-Hsu Yang, Chun-Ying Lin, Chia-Ling Chang, Bor-Luen Chiang

**Affiliations:** 1 Department of Pediatrics, Taipei City Hospital, Zhongxing Branch, Taipei, Taiwan; 2 Department of Pediatrics, National Taiwan University Hospital and National Taiwan University College of Medicine, No. 7, Zhongshan South Road, Zhongzheng District, Taipei, 100 Taiwan; 3 Department of Pediatrics, National Taiwan University Hospital, Hsin-Chu Branch, Hsin-Chu, Taiwan; 4 Department of Laboratory Medicine, National Taiwan University Hospital, Taipei, Taiwan

**Keywords:** Enthesitis, HLA-B27, Juvenile idiopathic arthritis, Sacroiliitis, Subtype

## Abstract

**Background:**

Juvenile idiopathic arthritis (JIA) has been categorized into seven different categories according to the International League of Associations for Rheumatology (ILAR) criteria. Enthesitis-related arthritis (ERA) was found to represent the largest category in a Taiwanese cohort study. The aim in this study was to compare the clinical characteristics, treatments, and outcomes of ERA in a single tertiary center in Taiwan, as compared to those of other categories of JIA. Furthermore, we determined patients’ characteristics and risk factors that can help assess the outcomes in ERA.

**Methods:**

A retrospective chart review of all patients with JIA referred to a pediatric rheumatology clinic in the National Taiwan University Hospital between 1993 and 2018 were identified according to ILAR criteria. Outcomes were assessed based on the Wallace criteria to categorize patients into active and non-active, including inactive, remission on medication, and remission off medication, groups. A subset of samples was further tested by DNA sequencing to identify HLA-B27 subtypes.

**Results:**

One-hundred and eighty-three patients were included in the study, with a mean of 8 years’ follow-up. ERA was the single largest category of JIA (39.9%); psoriasis and undifferentiated JIA were both the least common type (0.5%). ERA was male predominant (86%), had a late age of onset (11.0 ± 3.2 years), and the majority of ERA patients was HLA-B27-positive (97%). Of 25 HLA-B27-positive ERA patients checked by HLA-B27 sequencing, 23 were B*27:04 and 2 were B*27:05. ERA patients were significantly less likely to achieve non-active status compared to patients with persistent oligoarthritis (*P* = 0.036). In terms of treatment response to TNF-α inhibitors in methotrexate-refractory ERA, 26 patients remained active and only 11 patients (30%) achieved a non-active status. Sacroiliitis was a risk factor contributing to poorer treatment response in ERA (*P* = 0.006).

**Conclusion:**

ERA represented the most common category of JIA in Taiwan. Those ERA patients with sacroiliitis were likely to have persistent active disease and may require a more aggressive treatment strategy to improve their outcomes.

## Background

Juvenile idiopathic arthritis (JIA) is the most common chronic rheumatic disease in children; it describes a group of heterogeneous types of arthritis that begins before the age of 16 years and persists for more than 6 weeks with exclusion of other known conditions such as infection and malignancy [1]. The term JIA was defined by the International League of Associations for Rheumatology (ILAR), which has categorized it into seven categories based on the presentation in the first 6 months of illness. Among those categories, enthesitis-related arthritis (ERA) is an HLA-B27-related type of JIA characterized by the involvement of the entheses, peripheral joints, and the axial skeleton [2]. In most north American or European studies, oligoarthritis is the most common category among all JIA patients in contrast to ERA, which represents only 10–16% of all JIA patients [3–7]. Compared to other categories of JIA, children with ERA tend to have higher disease activity, greater pain intensity, and worse health outcomes [7]. It is also more difficult for ERA patients to achieve and maintain inactive disease than patients with other JIA categories [8].

However, the distribution of JIA categories in India or Taiwan varies greatly from that determined in studies in Western countries [9, 10]. Previously, a community-based JIA cohort study in Taiwan demonstrated that ERA represented the largest category among all Taiwanese JIA patients [9]. Nevertheless, few studies have analyzed the treatment outcomes, disease characteristics, and risk factors in Eastern ERA patients. Thus, the aim of this study was to compare the clinical characteristics, treatments, and outcomes of ERA to other categories of JIA in a single tertiary center in Taiwan. In addition, we sought to determine patients’ characteristics and risk factors that can help assess the development of active and non-active disease outcomes after treatment in patients with ERA. Lastly, we compared similarities and differences between patients from eastern and western countries.

## Patient and methods

This study involved a retrospective chart review of patients diagnosed with JIA between March 1993 and December 2018 at a pediatric rheumatology clinic in National Taiwan University Hospital (NTUH), Taipei, Taiwan. For all patients, demographic details, family history, diagnoses, disease onset age, onset joint sites and number, medication history, onset laboratory data, such as HLA-B27, antinuclear antibodies (ANA), and rheumatoid factor (RF) status, were routinely recorded. In addition, clinical or radiographic evidence of sacroiliitis was defined as patients suffering from low back pain for at least 3 months or have definite radiographic sacroiliitis that is based on the modified New York criteria or active (acute) inflammation on MRI [11, 12]. The JIA categories were categorized by the treating rheumatologist and were confirmed by the primary investigator using the ILAR classification criteria at 6 months after the onset of the disease. Disease onset age was defined as the date of the patient developed the clinical arthritis. The age at the follow-up visit was defined by the age of the patient at the last clinic visit. The medications used by each patient, including non-steroidal anti-inflammatory drugs (NSAIDs), oral prednisolone, intra-articular injection (IAI) of glucocorticoids, disease-modifying anti-rheumatic drugs (DMARDs), and TNF-α inhibitors were reviewed and recorded.

Treatment responses were evaluated based on the Wallace criteria at the last visit of clinic, according to which inactive disease was defined as follows: no active arthritis, no systemic symptoms attributable to JIA, no active uveitis, normal erythrocyte sedimentation rate and C-reactive protein level, and best possible physician’s global assessment on the scale used. Clinical remission on-medication was defined as meeting the criteria for inactive disease for a minimum of 6 continuous months while the patient was on medication. Clinical remission off-medication was defined as meeting the criteria for inactive disease for a minimum of 12 continuous months while off all anti-arthritis and anti-uveitis medications [13]. We combined inactive disease, clinical remission on-medication, and clinical remission off-medication groups into a non-active group to improve statistical analysis.

A subset of samples was further analyzed by HLA-B27 DNA sequencing. Whole blood samples were acquired in K3-ethylenediaminetetra-acetic acid tubes. DNA from whole blood stored at 4 °C was extracted with the MagCore Genomic DNA Whole Blood Kit (RBC Bioscience Corp., New Taipei City, Taiwan) according to the manufacturers’ instructions. The final DNA concentration was approximately 10–70 ng/ul. HLA-B sequence-based typing was performed using commercial polymerase chain reaction-sequence-based typing kits (HLAssure SE SBT Kit, Texas BioGene, Inc., Richardson, TX, USA). Cycle sequencing of exons 2, 3, and 4 of the HLA-B gene was performed using an Applied Biosystems 3730XL sequencer (Thermo Fisher Scientific Inc., Waltham, MA, USA) in both forward and reverse directions. Allele assignment was performed with AccuType HLA sequencing software (Texas BioGene, Inc.).

Statistical analysis was performed using SPSS Statics software (version 22; IBM Corp., Chicago, IL, USA). The differences between the active group and non-active group were analyzed using an unpaired Student’s *t*-test for continuous variables, the chi-squared test and Fisher’s exact test for categorical variables. The statistical significance was set at a *p* value < 0.05.

## Results

**Fig. 1:**
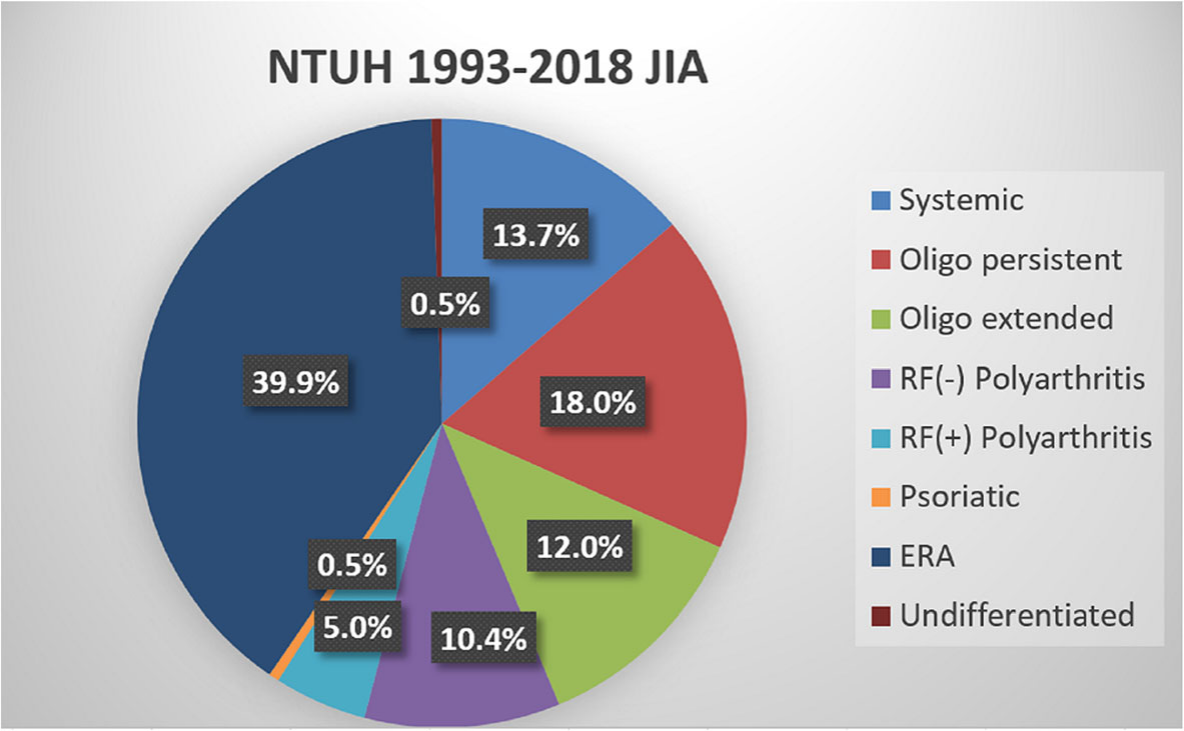
Distribution (%) of juvenile idiopathic arthritis (JIA) subtypes in 183 Taiwanese children

Figure 1 shows the distribution of JIA categories among 183 Taiwanese children. ERA represented the single largest category of JIA (39.9%), while psoriasis and undifferentiated JIA were both the least common category (0.5%). Psoriasis and undifferentiated categories were excluded from our further analysis; thus, 181 patients with a mean follow-up duration of 7.7 ± 5.9 years were included in the analysis. The demographic details of ERA patients are shown in Table 1. In the ERA patients, there was male predominance (86%); the onset tended to occur late (11.0 ± 3.2 years) and be pauciarticular (97%), and the majority of ERA patients were HLA-B27 positive (97%). Of ERA patients, 97% had arthritis or enthesitis involvement, and 16% had clinical or radiographic evidence of sacroiliitis. Only 10% of patients developed anterior uveitis. Twenty-five ERA patients who were positive for HLA-B27 underwent HLA-B27 sequencing, which revealed that 23 patients had the B*27:04 and 2 patients had the B*27:05 genotype.

**Table 1:** Enthesitis-related arthritis (ERA) patients’ and disease characteristics (*n* = 73)

Characteristics	*N* (%)
Demographic characteristics	
Male sex	63 (86)
Age at onset, mean ± SD years	11.0 ± 3.2
Age at follow-up visit, mean ± SD years	17.6 ± 5.4
Disease duration at follow-up visit, mean ± SD years	6.5 ± 5.0
Disease characteristics	
Enthesitis and arthritis	71 (97)
Presence of HLA-B27 antigen	67 (97^a^)
Clinical or radiographic evidence of sacroiliitis	12 (16)
Onset of arthritis in a male > 6 years old	68 (93)
Acute (symptomatic) anterior uveitis	7 (10)
History of AS/ERA/sacroiliitis with inflammatory bowel disease, Reiter’s syndrome, or acute anterior uveitis in a 1st degree relative	8 (11)
Pauciarticular (≤ 4 joints) onset	71 (97)
Genetic marker with HLA-B 27 subtypes^b^	
B*27:04	23 (92)
B*27:05	2 (8)

^a^only 69 patients underwent HLA-B27 testing

^b^only 25 HLA-B27-positive patients underwent subtype testing

**Fig. 2:**
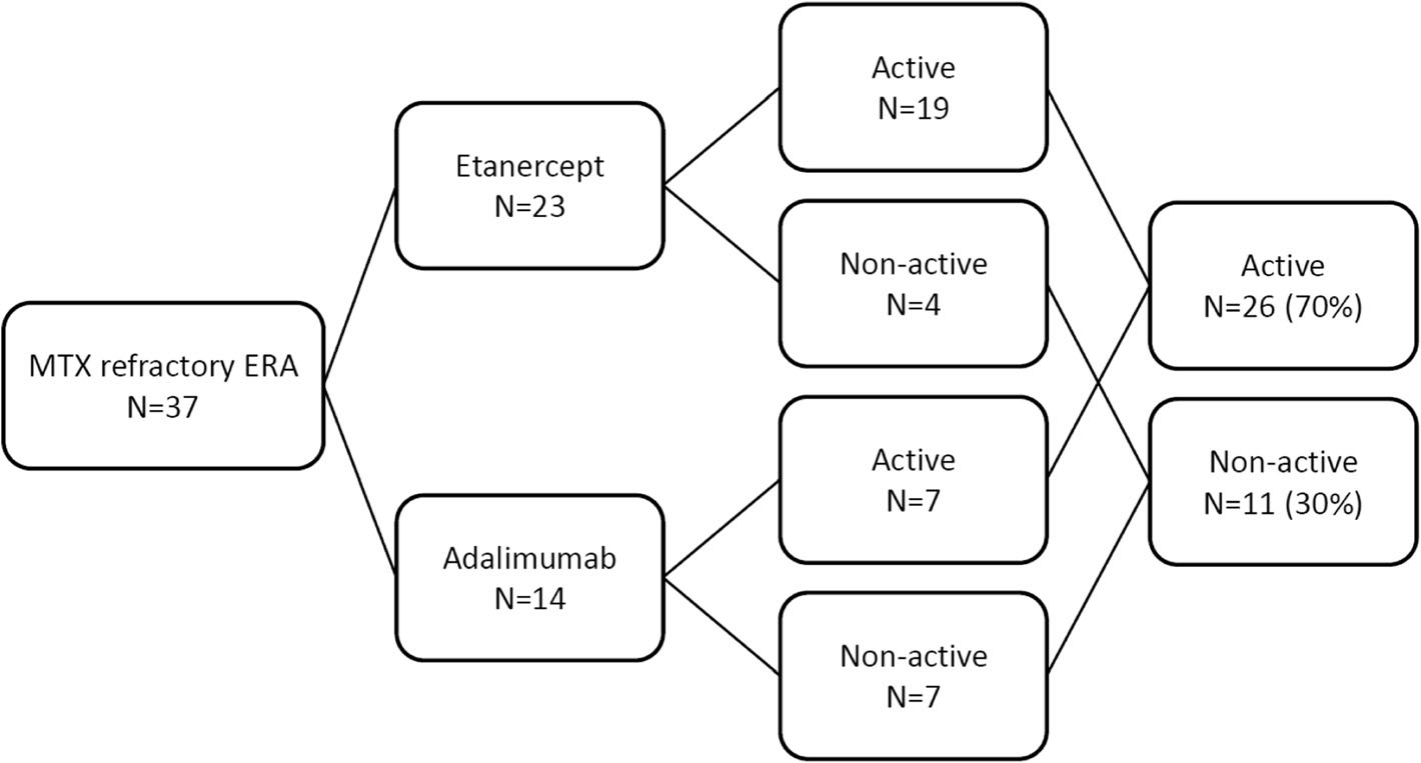
The effects of anti-TNFα in methotrexate-refractory enthesitis-related arthritis (ERA)

Medication history in ERA and other categories of JIA are shown in Table 2. NSAIDs were most used as first-line treatment in all categories (98%). Oral steroids were the least commonly used medication in ERA category, as compared to other categories of JIA. IAI of glucocorticoids was not commonly performed in our center. Only 4% of all JIA patients and 3% of persistent oligoarthritis had received this procedure. In ERA patients, the most commonly used conventional DMARD was methotrexate (MTX) (74%), followed by sulfasalazine (62%). In addition, 57 of 73 (78%) ERA patients in our cohort received TNF-α inhibitors. Of them, 37 patients were defined as MTX-refractory ERA, meaning that their disease status remained active under MTX treatment (10 mg/m^2^/week) for at least 3 months. Figure 2 shows that only 11 of 37 (30%) MTX-refractory ERA patients attained a non-active disease status after using TNF-α inhibitors. Another biological agent tocilizumab was used to treat systemic JIA patients. Eight systemic JIA patients in our series received tocilizumab treatment (data not shown in Table 2).

**Table 2:** Outpatient medication history for enthesitis-related arthritis (ERA) and other subtypes of juvenile idiopathic arthritis (JIA)

*N* (%)	ERA	Persistent Oligoarthritis	Extended Oligoarthritis	RF(+) Polyarthritis	RF(−) Polyarthritis	Systemic	Overall
	*n* = 73	*n* = 33	*n* = 22	*n* = 9	*n* = 19	*n* = 25	*n* = 181
NSAIDs	70 (96)	33 (100)	22 (100)	9 (100)	19 (100)	24 (96)	177 (98)
Oral glucocorticoids	39 (53)	20 (61)	18 (82)	7 (78)	16 (84)	18 (72)	118 (65)
Intra-articular glucocorticoid injection	4 (5)	1 (3)	1 (5)	1 (11)	0 (0)	0 (0)	7 (4)
Methotrexate	54 (74)	20 (61)	17 (77)	6 (67)	16 (84)	17 (68)	130 (72)
Sulfasalazine	45 (62)	11 (33)	15 (68)	4 (44)	10 (53)	7 (28)	92 (51)
Azathioprine	24 (33)	18 (55)	16 (73)	4 (44)	10 (53)	16 (64)	88 (49)
Colchicine	2 (3)	0 (0)	0 (0)	0 (0)	0 (0)	1 (4)	3 (2)
Cyclosporine	1 (1)	2 (6)	2 (9)	1 (11)	1 (5)	3 (12)	10 (6)
Plaquenil	12 (16)	7 (21)	6 (27)	6 (67)	3 (16)	3 (12)	37 (20)
TNF-α inhibitor	57 (78)	19 (58)	18 (82)	5 (56)	17 (89)	17 (68)	133 (73)

Table 3 shows the category-specific outcomes of each category; persistent oligoarthritis had the best treatment outcome, with 55% able to achieve non-active disease status. Thus, compared to the best treatment outcome category, ERA had a worse prognosis, with 33% (*P* = 0.036) able to achieve non-active disease status.

**Table 3:** Juvenile idiopathic arthritis category-specific outcomes

	ERA	Persistent Oligoarthritis	Extended Oligoarthritis	RF(+) Polyarthritis	RF(−) Polyarthritis	Systemic	Overall
	*n* = 73	*n* = 33	*n* = 22	*n* = 9	*n* = 19	*n* = 25	*n* = 181
Active	46 (63)	14 (42)	15 (68)	7 (78)	10 (53)	14 (56)	106 (59)
Non-active	24 (33)	18 (55)	5 (23)	2 (22)	8 (42)	8 (32)	65 (36)
Inactive	13 (18)	2 (6)	3 (14)	2 (22)	3 (16)	0 (0)	23 (13)
Clinical remission, on-medication	5 (7)	1 (3)	0 (0)	0 (0)	0 (0)	0 (0)	6 (3)
Clinical remission, off-medication	6 (8)	15 (46)	2 (9)	0 (0)	5 (26)	8 (32)	36 (20)
Lost to follow-up	3 (4)	1 (3)	2 (9)	0 (0)	0 (0)	2 (8)	8 (4)
Expired	0 (0)	0 (0)	0 (0)	0 (0)	1 (5)	1 (4)	2 (1)
Active/Non-active (P)	46/24 (^*^0.036)	14/18 (reference)	15/5 (^*^0.027)	7/2 (0.071)	10/8 (0.422)	14/8 (0.151)	106/65

^*^In comparison to persistent oligoarthritis. *P* < 0.05 is statistically significant

The risk factors contributing to poor outcomes in ERA are shown in Table 4, based on a comparison between ERA patients with active vs non-active disease. Sacroiliitis patients were more likely to suffer from persistent active disease.

**Table 4:** The risk factors of active disease in enthesitis-related arthritis (*N* = 70)

	Active *n* = 46	Inactive *n* = 13	*P*	Non-active^a^ *n* = 24	*P*
≤4 joints involved	44	13	> 0.999	24	0.543
> 4 joints involved	2	0	> 0.999	0	0.543
Anterior uveitis	7	0	0.330	0	0.087
prednisolone used, ever	27	7	0.762	13	0.801
Family history of HLA-B27-related disease	3	2	0.303	5	0.113
HLA-B27-positivity	42	12	> 0.999	23	0.654
Clinical or radiographic evidence of sacroiliitis	12	0	0.051	0	0.006
Hip arthritis	12	5	0.491	8	0.583
B*27:04^b^	14	9	> 0.999	–	–
B*27:05^b^	1	1	> 0.999	–	–

^a^Non-active: Inactive + Remission on/off medication

^b^only 25 HLA-B27-positive patients underwent subtype testing

## Discussion

In this study, we compared the clinical characteristics, treatments, and outcomes of ERA in Taiwan, as compared to other categories of JIA and sought to identify risk factors that can help to assess the outcomes in ERA, given the differences in epidemiology of JIA in Western and Eastern JIA patients, and the paucity of such studies in Eastern JIA patients. We found that ERA was the most common JIA category in Taiwan, showed a male preponderance (86%), had a late age of onset (11.0 ± 3.2 years), and was more likely to involve HLA-B27-positivity (97%). ERA patients tended not to achieve non-active status, and sacroiliitis presented a poorer treatment response in ERA patients (*P* = 0.006).

Epidemiological studies of Western JIA patients has revealed oligoarthritis as the most common category, with a prevalence of 27–56%, compared to that of ERA of only 3–11% [1, 14]. However, ERA was the most common category of JIA in Chinese or non-Western populations, with prevalence of 36–37.4% [9, 10]. Similar epidemiology result was observed in a study conduct in a multiethnic city Toronto, Canada, which has examined the distribution of JIA categories from patients with different ethnic origins. Their results showed ERA patients with Asian origin accounted the most predominant group (24%), followed by Indian subcontinent (12.5%), Arab (11.8%), black (9.7%) and European descendant accounted for the least (7.6%) in ERA [15]. Few previous ERA cohort studies have analyzed the disease characteristics, treatments, and outcomes in a non-Western country, prompting us to investigate whether the prognosis and disease characteristics were similar between Eastern and Western populations. According to ILAR diagnostic criteria, most our cases could be well categorized. Only 0.5% of the patients were classified as undifferentiated arthritis. Furthermore, psoriatic arthritis also accounted only 0.5% of the patients. Yen, et al. reported a childhood and adolescent psoriasis estimated prevalence of 0.09% in Taiwan [16], which was comparable to the results of another study using Taiwan’s National Health Insurance (NHI) database and was much lower than the reported prevalence of 0.7–6.2% at other countries [17, 18]. Yen also found 14% of children and adolescent with psoriasis developed arthritis [16]. Combined, it may explain why psoriatic arthritis was the least common JIA category in Taiwan.

A recent multicenter ERA cohort study performed in the United States enrolled 234 children; there was also a male preponderance (72.2%), a median onset age of 11.6 years, 59.2% HLA-B27-positivity, and pauciarticular onset (78%) [19]. These results are similar to our findings in terms of the age of onset, sex distribution, and number of joints affected at onset. However, the HLA-B27-positivity in the previous study was lower than in our study, which might be attributed to ethnic variation [15, 20]. HLA-B27 subtype sequencing results only revealed that most of our patients carried the HLA-B*2704 genotype, which is compatible with their Asian ethnicity. This result was similar to those of previous studies [21, 22]. Additionally, our findings revealed that HLA-B27 polymorphism had a minor influence on clinical phenotypes (data not shown), similar to other previous studies conducted from different countries [22–27].

Since ERA is one of JIA category disease, the treatment strategy is based on the treatment recommendation or consensus guidelines for JIA, which favors NSAIDs as first line medication and DMARDs as second-line medication [28, 29]. In the DMARDs selection, MTX is recommended as the prior medication for peripheral arthritis but not for active sacroiliitis [29]. Moreover, ERA is included into juvenile spondyloarthropathy (JSpA), which is a prototypical ankylosing spondylitis (AS) [30]. Thus, ERA treatment regimens can also be based on the treatment recommendation for adult AS, in which sulfasalazine is used as the conventional DMARD choice [31]. Therefore, the ideal conventional DMARDs chosen for the treatment of ERA patients are MTX for peripheral arthritis and sulfasalazine for axial involvement; these medication choices were also observed in our center’s ERA patients. Most of our ERA patients presented initially with peripheral arthritis, so they were treated with MTX first. Later on, some were refractory to MTX alone and/or developed active sacroiliitis would further be prescribed with sulfasalazine. Only 16% of ERA patients had clinical or radiographic evidence of sacroiliitis may also contribute to the lower using rate (62%) of sulfasalazine than MTX (74%). Few patients in our study received IAI of glucocorticoids, although such treatment is recommended as an effective therapy for JIA, especially oligoarthritis [28, 29]. The reason was that in our center, the procedure of echo-guided IAI did not well developed until 3 years ago. The number of case receiving IAI of glucocorticoid is now increasing gradually.

Furthermore, in the recent decade, biological agents, such as TNF-α inhibitors, have provided another effective and safe treatment options for various autoimmune diseases, including JIA, but their expensive costs have limited their application. According to the NHI payment guideline in Taiwan, TNF-α inhibitors could be used as the first line biological agent for JIA if the patient has tried MTX (10 mg/m2/week) for at least 3 months without improvement. Therefore, this policy lead to a higher percentage of Taiwanese ERA patients chose MTX as their DMARDs to control the disease.

As compared to other categories of JIA, the ERA category has the tendency to remain persistent active disease. In this respect, there was no difference between Eastern and Western studies. One referral-based cohort in a German study that analyzed the long-term outcome of JIA found that ERA patients have a very low remission rate (18% compared to 54% for oligoarticular patients) [32]. Our study revealed a similar finding, with a 33% remission rate for the ERA category as compared to a 55% remission rate for persistent oligoarthritis. A concept of early aggressive treatment in JIA during the window of opportunity was proposed to change the biology of the disease and improve long-term disease outcomes [33]. The effectiveness of early treatment by biological agents were observed in systemic JIA and polyarticular JIA treated by anti-IL 1 and infliximab with methotrexate respectively [34, 35]. However, the cost/efficacy benefit of early biologics treatment in ERA is still less clear. A prospective observational study conducted from the Dutch Arthritis and Biologicals in Children Registry has evaluated the effect of TNF inhibitors which has shown although 73% of patients achieved ACR Pediatric 70 improvement within three months but ERA patients were still unable to attain a sustained disease-free state [8]. In ERA patients, the disease tended to remain active even after MTX or TNF-α inhibitor treatment, in comparison with other categories of JIA. One study evaluated 125 patients’ disease status after concurrent treatment with MTX and a TNF-α inhibitor. In category-specific outcomes, compared to RF-negative polyarthritis, ERA patients were less likely to have inactive disease at the 1-year follow-up (57% vs 24%; *p* = 0.02) and to ever attain inactive disease status (76% vs 43%; *p* = 0.03) [36]. In addition, a similar poorer treatment outcome in the ERA category was reflected in the more severe patient groups, such as those that are MTX-refractory. In our MTX-refractory ERA category, after TNF-α inhibitor treatment, only 30% attained a non-active disease status; this was lower than in the non-ERA MTX-refractory polyarthritis JIA (remission group = 52%) from our previous study [37].

Other studies of risk factors contributing to poorer treatment outcome in ERA patients revealed no differences between Eastern and Western analyses. One study from Norway investigated predictors of failure to achieve remission and development of sacroiliitis, and showed that these factors included AS in a first-degree relative, the presence of HLA–DRB1*08, and ankle arthritis within the first 6 months of disease [38]. Furthermore, an 8-year follow-up Nordic population-based JIA cohort study indicated that HLA-B27-positivity, any clinical signs of sacroiliitis, enthesitis, and hip arthritis predicted a more chronic disease course [39]. Our study revealed that any clinical signs of sacroiliitis had the most significant influence on a poorer treatment response in ERA patients.

Our study had several limitations. First, this was a retrospective, single-center observational study with limited case numbers. Nonetheless, this represented one of the largest cohorts studied in Taiwan. Our hospital, NTUH is a referral center located in Taipei city. It provides services for patients mainly from northern Taiwan including Taipei city, New Taipei city, Keelung city, Yilan county, part of Taoyuang city and Hsinchu city/county with an estimated pediatric population more than 3.6 million. Since JIA is identified as catastrophic illness in Taiwan, most patients will be transferred to referral center for long-term treatment and follow-up. However, there may be still some very mild JIA cases that are treated successfully at regional hospitals. This might contribute some selection bias in our study. Second, the time-to-event variables had a large standard deviation, with mean follow-up duration of 6.5 ± 5.0 years in the ERA patients. Thus, a patient enrolled earlier into our study had a longer follow-up duration, with more complete follow-up information, than patients with a shorter follow-up duration. Different follow-up durations might result in the true treatment responses for each category of JIA being unknown. Third, the small sample size in our ERA follow-up group (*N* = 70, active = 46, non-active = 24) might have influenced our risk factor analysis, in which only one factor had a significant impact on persistent active disease in ERA patients. Lastly, since the number of patient who underwent HLA-B27 subtyping is small (*N* = 25, B*27:04 = 23, B*27:05 = 2), that may mislead to the conclusion that HLA-B27 polymorphism has minor impact on clinical phenotype and treatment outcomes.

## Conclusion

ERA has similar characteristics, treatments, and outcomes between Western countries and Taiwan, except that it is the more predominant JIA category in Eastern JIA category analysis. Among the seven categories of JIA, ERA had a worse treatment response with a tendency to retain active disease status, even after TNF-α inhibitor treatment. Moreover, ERA patients with sacroiliitis warrant a more aggressive treatment strategy during the window of opportunity, which might improve the overall treatment response in this category of JIA.

## Data Availability

The data are available on request to the corresponding author.

## Abbreviations

ANA: Anti-nuclear antibody
AS: Ankylosing spondylitis
DMARD: Disease-modifying anti-rheumatic drug
ERA: Enthesitis-related arthritis
IAI: Intra-articular injection
ILAR: International League of Associations for Rheumatology
JIA: Juvenile idiopathic arthritis
JSpA: Juvenile spondyloarthropathy
MTX: Methotrexate
NHI: National Health Insurance
NSAID: Non-steroidal anti-inflammatory drug
NTUH: National Taiwan University Hospital
PCR: Polymerase chain reaction
RF: Rheumatoid factor
